# The impact of adolescents’ health motivation on the relationship among mental stress, physical exercise, and stress symptoms during COVID-19: A dual moderation model

**DOI:** 10.3389/fpubh.2023.1164184

**Published:** 2023-04-11

**Authors:** Hu Lou, Jin Chen, Ping Liu

**Affiliations:** School of Sports Science, Nantong University, Nantong, Jiangsu, China

**Keywords:** adolescent mental stress events, stress symptoms, physical exercise, mental stress buffer, health motivation

## Abstract

**Objective:**

Many Chinese teenagers are experiencing high mental stress levels due to epidemic-related restrictions and closures. Mental stress can induce numerous associated symptoms, and physical exercise is considered to buffer mental stress. However, it remains unclear whether health motivation regulates the relationships among mental stress, physical exercise, and stress symptoms. This study examined whether mental stress events during the epidemic can predict stress symptoms, whether physical exercise can buffer mental stress, and whether the mental stress buffer effect is enhanced when health motivation regarding physical exercise is high.

**Methods:**

In total, 2,420 junior high school students (1,190 boys and 1,230 girls; 826 seventh-grade students, 913 eighth-grade students, and 681 ninth-grade students) from nine provinces nationwide were selected to investigate mental stress events, symptoms, health motivation, and physical exercise in adolescents. The hypothesis was tested with a multiple regression analysis.

**Results:**

A positive relationship between adolescent mental stress events and stress symptoms was observed, and an interactive relationship was found among health motivation, physical exercise, and mental stress factors. Specifically, the mental stress-buffering effect of physical exercise was significant only when health motivation was high.

**Conclusion:**

In the post-epidemic period, the influence of mental stress events on stress symptoms in adolescents was found to be buffered by physical exercise only in terms of high health motivation. This result highlighted the role of health motivation in the buffering effect of physical exercise on mental stress during an epidemic.

## 1. Introduction

The early-2020 novel severe acute respiratory syndrome coronavirus 2 (SARS-CoV-2) outbreak and resulting coronavirus disease 2019 (COVID-19) prevalence represented a serious crisis and challenge in China. Major achievements have been made in combating this epidemic through the efforts of the population. Strategies for the prevention of COVID-19 and control of SARS-CoV-2 in China have recently changed from emergency to normal procedures (1). However, the epidemic resulted in substantial losses across the population. The long-term restrictions and closures had far-reaching effects on quality of life and productivity. Infected individuals have been reported to experience various sustained symptoms, even death in some cases. These negative events of the epidemic may represent strong mental stressors in the post-epidemic period, further inducing anxiety, depression, insomnia, post-traumatic stress disorder, and other mental disorders (2). Adolescents are in a period of growth and development, experience wide emotional fluctuations, and are highly susceptible to mental stress. Recently, academic mental stress in teenagers has become a relatively serious concern; thus, it is likely that the combined effect of the epidemic and academic stress has induced serious mental stress in this population. Improving the stress responses of teenagers is critical in the post-epidemic period.

Stress is defined as an environment or situation that threatens, challenges, or harms an individual’s physical and mental health. The body can adapt to stress; however, when the stimulus intensity is large or sustained, emotional problems such as anxiety and depression can arise, in addition to physical problems such as muscle tension, respiratory symptoms, and serious diseases (3). Not all individuals exhibit the same stress symptoms. A third variable exists between stress symptoms and stress events that can buffer stress: physical exercise can improve immunity, boost the mental state, antagonize mental stress, and buffer mental stress in young people in the post-epidemic period (3).

## 2. Literature review

Previous studies have focused on the buffering effect of physical exercise on stress symptoms caused by mental stress events from four main aspects (4). First, individuals who regularly participate in physical exercise exhibit a reduced sensitivity to mental stress events, and physical exercise can reduce mental stress. Second, physical exercise can strengthen individual and social resources, such as self-efficacy and social support, thus reducing mental stress. Third, physical exercise affects physiological and psychological reactions to stress, including stress-induced symptoms, anxiety, and cortisol release. Fourth, physical exercise can directly enhance physical health and compensate for the effects of mental stress.

Evidence shows that physical exercise can effectively buffer mental stress. Hauland et al. (5) investigated 1,577 adolescents aged 11 to 15 years and found that physical exercise provided a buffer against the health problems caused by academic stress. Sigfusdottir et al. (6) surveyed 7,232 junior high school students and found that physical exercise can buffer the impact of family conflict on depression. Gerber et al. (7) showed that physical activity could moderate the relationship between stressful events and quality of life.

However, the existing conclusions are inconsistent, and not all the evidence supports the positive mental stress buffer effect of physical exercise (8). This conflict may arise from a regulatory variable between physical exercise and mental stress buffering (9). Physical exercise motivation is the psychological motivation to participate in physical exercise. Different types of motivation are related to the physical and psychological effects of physical exercise. Therefore, the mental stress buffer effect of physical exercise may be regulated by motivation. Jsoard-Gautheur et al. (10) found that different motivation types have different mental stress buffering effects regarding physical exercise, which can alter the relationship between stress and psychological burnout. Health motivation is a type of motivation for physical exercise whereby individuals are motivated to exercise for health purposes. Individuals motivated by health are more likely to accept and participate in health-related interventions (11). Because people focused on health during the epidemic, health motivation may be a moderator of the mental stress buffer effect of physical exercise in this setting. However, the role of health motivation in the mental stress buffering effect of physical exercise during the epidemic has not yet been investigated.

Notably, motivation can significantly affect subjective feelings and behavior, and individuals are more inclined to be motivated to avoid loss and achieve personal goals (12). Health motivation is a desire to improve personal health and quality of life through physical exercise (13). Individuals motivated by health are more likely to accept and participate in physical exercise due to the perceived health benefits and are more likely to adopt behavioral intervention measures to promote health (14). Health motivation is a crucial component of the mental stress buffering effect of physical exercise during an epidemic and may be the key variable influencing this effect in adolescents.

In summary, motivation is closely related to the mental stress buffering effect of adolescent physical exercise. Especially during an epidemic, health motivation may play a regulatory role in the relationship between physical exercise and mental stress buffering. However, there is a lack of empirical research on the relationships among adolescent health motivation, physical exercise, mental stress events, and stress symptoms during the current epidemic. The following hypotheses were developed based on the literature reviewed above.

*H1*: Mental stress events during the epidemic period positively affected the stress symptoms of adolescents.

*H2*: Physical exercise can buffer the positive correlation between mental stress events and symptoms.

**Figure 1:**
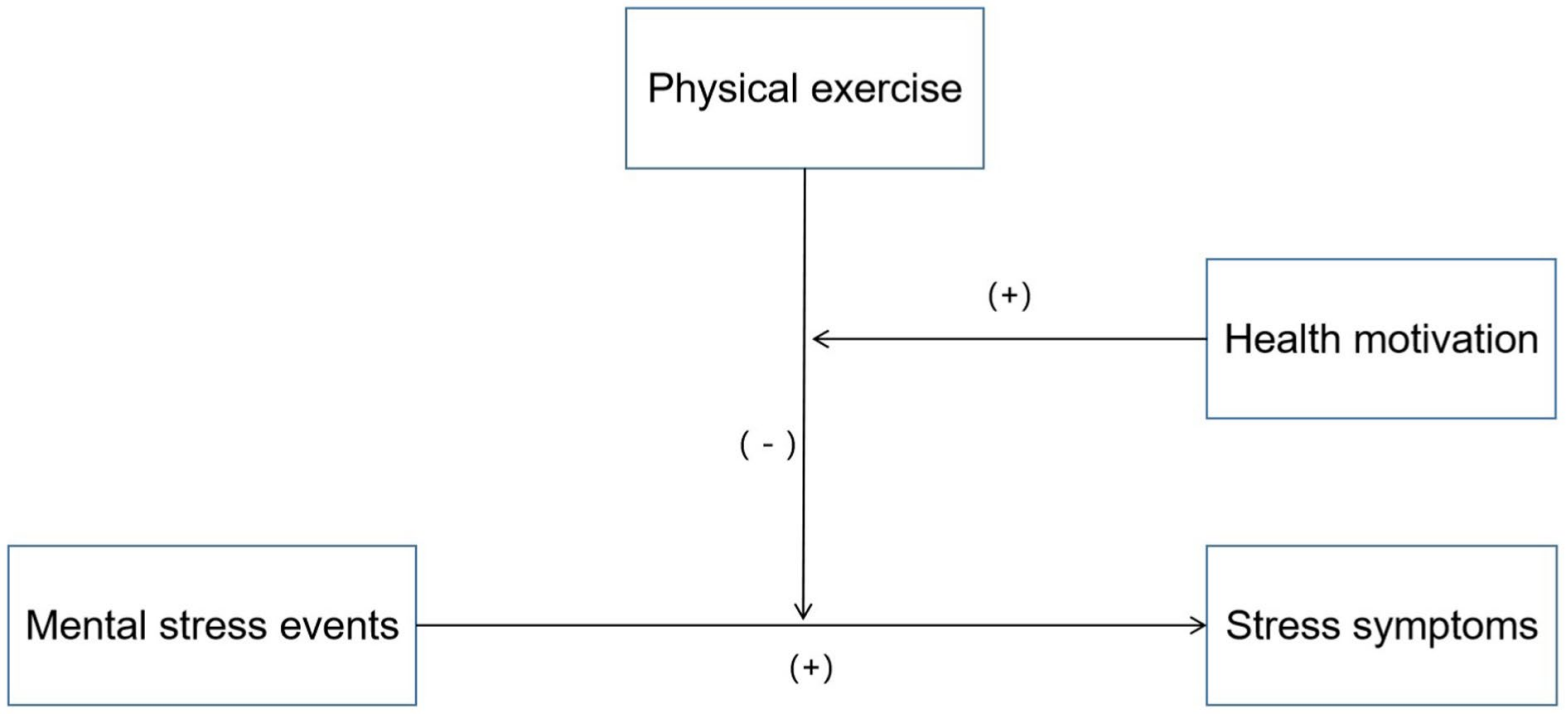
Hypothesis model diagram.

*H3*: The buffering effect is stronger if teenagers are motivated by health to engage in physical exercise. The hypothetical model is shown in Figure 1.

## 3. Methods

### 3.1. Sampling method and survey object

Participants were recruited from March to April 2022 from the three economic belts in the east, west, and middle of China. One city with a high GDP level, one with a medium GDP level, and one with a low GPD level was selected from each economic belt (east: Nantong, Jiangsu, Lishui, Zhejiang, Fuxin, Liaoning; west: Chongqing, Yibin, Sichuan, Ili, Xinjiang Uygur Autonomous Region; central: Taiyuan, Shanxi, Puyang, Henan, Xiangxi Tujia and Miao Autonomous Prefecture, Hunan). One junior high school was selected in each of the nine cities. The stratified random cluster sampling method was employed, and local teachers were entrusted to distribute 100 paper questionnaires per grade, totaling 2,700; 2,679 questionnaires were recovered, and 259 were invalid and excluded, leaving 2,420 effective questionnaires, representing an effective recovery rate of 89.6%.

### 3.2. Measuring scale

#### 3.2.1. Adolescent stress events

The Adolescent Self-rating Life Events Checklist (ASLEC), revised by Xin (15), was used to assess the recent stress events experienced by adolescents. This scale adopts a 6-point Likert scale scoring method, with “0” representing “never happened” and “5” representing “great impact.” The higher the score was, the more serious the event’s impact was. The scale includes five dimensions and a total of 26 items, four focused on interpersonal stress, four focused on learning stress, seven focused on punishment, six concerning loss dimensions, and five related to adaptation dimensions. The overall internal consistency coefficient was Cronbach *α* = 0.775, and the internal consistency coefficient of each subscale was Cronbach *α* = 0.711–0.854.

#### 3.2.2. Adolescent stress symptoms

We adopted the Calgary Symptoms of Stress Inventory (C-SOSI) compiled by Carlson (16). Two psychology graduate students translated the questionnaire. One international student whose native language is English translated the Chinese questionnaire back into English, and two psychology professors proofread and modified the Chinese and English questionnaires to form the Chinese version. The questionnaire included eight dimensions and a total of 56 items, including eight related to depression symptoms, seven concerning anger symptoms, nine focused on cross-nerve arousal, six regarding nervous symptoms, eight related to muscle tension symptoms, six focused on cardiopulmonary symptoms, six related to cognitive disorder symptoms, and six regarding upper respiratory tract symptoms. A 5-point Likert scale scoring method was applied, where “0” indicated “never,” and “4” indicated “very frequent.” The higher the score was, the more pronounced the stress symptoms of the participants were. The overall internal consistency coefficient was Cronbach *α* = 0.941, and the internal consistency coefficient of each dimension was Cronbach *α* = 0.711–0.873.

#### 3.2.3. Adolescent physical exercise

The adolescents’ physical exercise levels were evaluated using the Physical Activity Rating Scale (PARS) (17), which includes three items: the frequency of weekly physical exercise, the duration of each physical activity, and the intensity of regular physical exercise. The total physical exercise score is the product of the three items. Each item is divided into five grades with a scoring range of frequency and intensity of 1–5, a scoring range of time of 0–4, and a total score range of 0–100. The higher the value was, the higher the teenagers’ physical exercise levels were. Scores of 20–42 indicated moderate exercise levels, and ≥ 43 represented high exercise levels. The scale had high reliability and validity, retest reliability *r* = 0.82, and internal consistency reliability Cronbach *α* = 0.907.

#### 3.2.4. Adolescent health motivation

Adolescents’ health motivation was measured using the health motivation dimension in the Simplified Exercise Motivation Scale (MPAM-R) (18). There were three items in total. A 5-point Likert scale scoring method was used, where “1” represented “none,” and “5” represented “very strong.” The higher the score was, the higher the adolescents’ health motivation was. The internal consistency coefficient of the scale was Cronbach *α* = 0.714.

### 3.3. Data analysis

SPSS 25.0 and AMOS 24.0 were used for the statistical analysis of relevant data, and AMOS 24.0 was used for the confirmatory factor analysis of the measuring scale. SPSS 25.0 was used to standardize the data. Standard method deviation tests, descriptive statistical analyses, Pearson correlation analyses, linear regression analyses, Bootstrap adjustment effect analyses, and simple slope tests were performed, with *p* < 0.05 as the significance standard.

## 4. Results

### 4.1. Common method deviation test

Harman’s single-factor test was used to test the common method deviation in the data. Two factors with a feature root >1 were extracted, accounting for 70.49% of the total variance cumulatively. The first common factor accounted for 38.45% of the total variance; because this value was lower than 40% of the critical value, no serious common method deviation problems were present.

### 4.2. Description of subjects

Surveys were collected from 1,190 boys and 1,230 girls; thus, there was no sex imbalance present. There were 826 seventh-grade students, 913 eighth-grade students, and 681 ninth-grade students. There was no significant difference in the distribution of different grades (*X^2^* = 3.74, *p* = 0.15).

### 4.3. Correlation analysis of variables

The correlation analysis of the research variables (see Table 1) showed that total physical exercise was significantly negatively correlated with mental stress events and stress symptoms in adolescents. There was a significant negative correlation between health motivation and adolescent mental stress events and symptoms. Adolescent mental stress events and stress symptoms were significantly positively correlated, and the relationship between these variables satisfied the follow-up hypothesis.

**Table 1:** Correlation analysis of various variables.

	M ± SD	PARS	MSE	HM	SS
PARS	40.67 ± 6.85	1			
MSE	26.85 ± 15.55	−0.040*	1		
HM	9.70 ± 2.78	0.339**	−0.080**	1	
SS	63.89 ± 28.61	−0.075*	0.479**	−0.058**	1

*p < 0.05; **p < 0.01; PARS, adolescent total physical exercise; MSE, mental stress events; HM, health motivation; SS, stress symptoms.

### 4.4. Simple regression effect analysis

A regression effect analysis was conducted using the input method with adolescent mental stress events, total physical exercise, and health motivation as independent variables and stress symptoms as the dependent variable. The results revealed that adolescent mental stress events could positively affect stress symptoms (*β* = 0.478, SE = 0.033, *p* < 0.001), supporting Hypothesis 1. However, total physical exercise and health motivation did not directly affect stress symptoms (*p* > 0.05, see Table 2).

**Table 2:** Linear regression analysis of adolescent mental stress events and stress symptoms.

	*β*	SE	*t*	*p*
Constant		3.347	13.096	*p* < 0.001
MSE	0.478	0.033	26.665	*p* < 0.001
PARS	−0.012	0.079	−0.624	*p* = 0.533
HM	−0.015	0.195	−0.802	*p* = 0.423

MSE, mental stress events; PARS, adolescent total physical exercise; HM, health motivation.

### 4.5. Buffering effect of health motivation and exercise on mental stress

#### 4.5.1. Buffering effect of exercise on mental stress

Model 3 in Process was used, with health motivation and physical exercise as the adjusting variables, adolescent mental stress events as the independent variable, and adolescent stress symptoms as the dependent variable. The results showed that the interaction items of physical exercise and mental stress events did not affect stress symptoms (*p* = 0.305), and that this interaction item had no significant effect on stress symptoms when distinguishing between high and moderate exercise levels (*P_H_* = 0.715, *P_M_* = 0.511). Thus, physical exercise cannot buffer mental stress, and Hypothesis 2 is rejected (see Table 3).

**Table 3:** The buffering effect of health motivation and exercise on stress.

Variables	PARS				PARS_H_				PARS_M_			
	*β*	SE	*t*	*p*	*β*	SE	*t*	*p*	*β*	SE	*t*	*p*
Constant	−0.001	0.019	−0.043	0.965	−0.006	0.036	−0.157	0.876	0.040	0.025	1.590	0.112
MSE	0.426^**^	0.021	22.470	0.000	0.611^**^	0.034	17.755	0.000	0.533^**^	0.032	16.741	0.000
PARS	−0.019	0.022	−0.869	0.385	−0.066	0.040	−1.633	0.103	−0.049	0.028	−1.756	0.079
HM	−0.045^*^	0.022	−2.106	0.035	−0.114^**^	0.029	−3.892	0.000	−0.062^*^	0.026	−1.986	0.024
MSE × PARS	−0.022	0.021	−1.025	0.305	−0.014	0.037	−0.365	0.715	−0.020	0.031	−0.658	0.511
MSE × HM	−0.010	0.020	−0.498	0.618	0.033	0.037	0.912	0.362	−0.007	0.034	−0.202	0.840
PARS×HM	0.004	0.022	0.186	0.853	0.002	0.036	0.059	0.953	−0.044	0.028	−1.551	0.121
MSE × PARS×HM	0.013	0.020	0.657	0.511	−0.040	0.033	−1.228	0.220	0.056^*^	0.029	2.133	0.015
*R* ^2^	0.232				0.348				0.219			
*F*	103.869				61.095				64.086			

*p < 0.05; **p < 0.01; PARS, adolescent total physical exercise; PARS_H_, means high exercise level; PARS_M_, means moderate exercise level; MSE, means mental stress events; HM, means health motivation.

#### 4.5.2. Regulatory effect of health motivation on the mental stress buffering effect of exercise

According to the results shown in Table 3, the interactions between adolescent health motivation and adolescent physical exercise and between adolescent health motivation and adolescent mental stress events do not buffer stress symptoms, even when different exercise levels are considered (*p* > 0.05). The interaction between health motivation, mental stress events, and physical exercise has no buffering effect on stress symptoms; however, when different exercise levels are considered, health motivation regulated the mental stress buffering effect among those engaging in moderate (*p* < 0.01), but not high, exercise levels (see Table 3).

**Figure 2:**
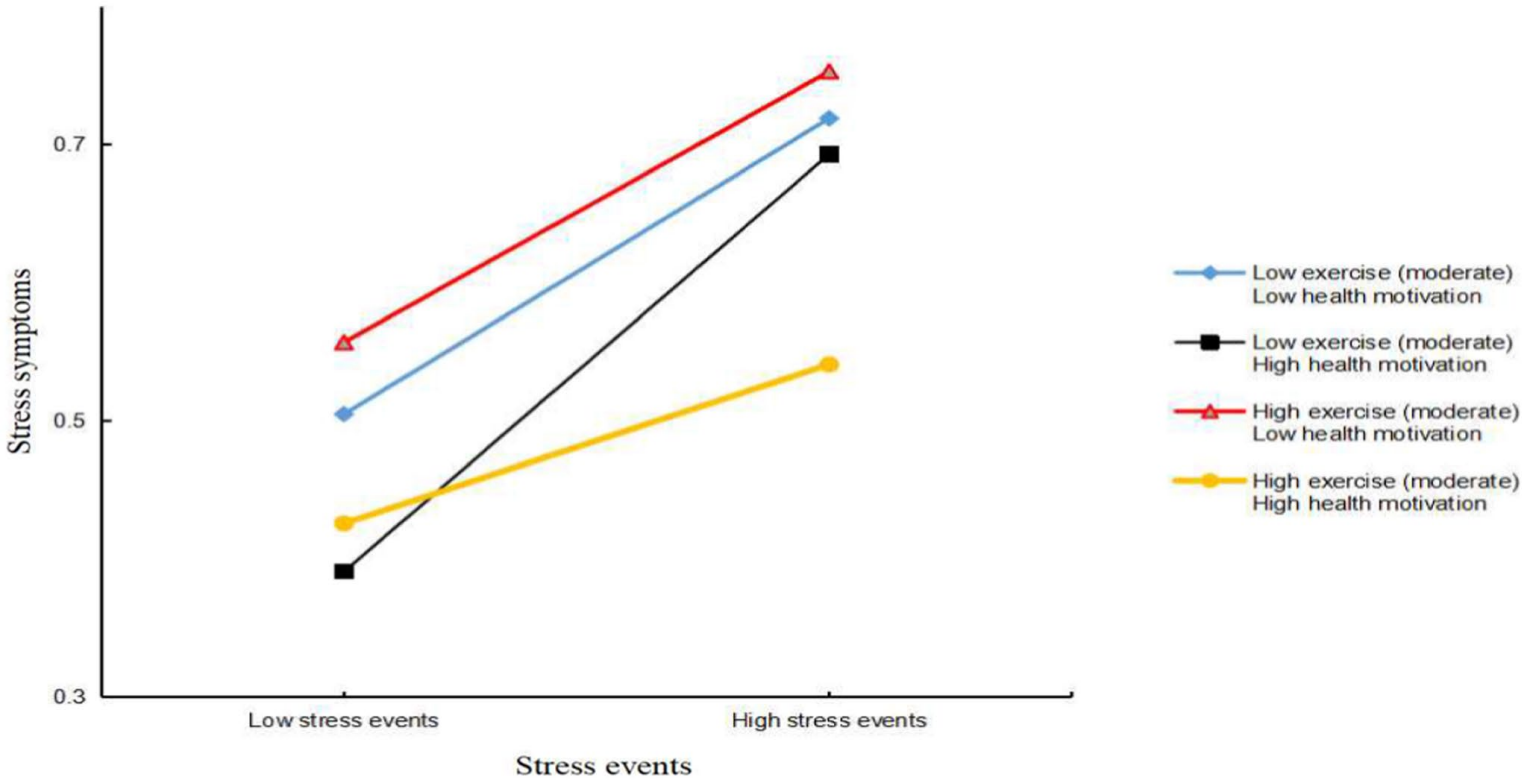
Simple slope analysis of health motivation to moderate exercise level stress buffer.

Furthermore, through a simple slope analysis, we explored how health motivation regulates the buffering effect of moderate exercise on mental stress (see Figure 2). The results revealed no regulatory effect of health motivation, regardless of the extent of their motivation, among adolescents engaging in a low level of moderate exercise (M-1SD); however, among adolescents engaging in a high level of moderate exercise (M + 1SD), increased health motivation increased the buffering effect.

## 5. Discussion

This study investigated physical exercise, health motivation, mental stress events, and stress symptoms in adolescents during the epidemic, confirmed that physical exercise can mediate the relationship between mental stress events and stress symptoms in adolescents, and revealed that health motivation can strengthen the mental stress buffering effect of physical exercise during an epidemic.

### 5.1. Mental stress buffering effect of adolescent physical exercise during the epidemic

Consistent with previous findings (19), our survey revealed that mental stress events in adolescents positively affected stress symptoms during the epidemic, supporting Hypothesis 1. This result provided a premise for further investigation into the buffering effect of physical exercise on mental stress effects.

The results showed that the interaction between physical exercise and mental stress events had no significant effect on stress symptoms, and the distinction between high and moderate physical exercise volumes was also not significant, indicating that physical exercise did not buffer the mental stress of adolescents during the epidemic. Thus, Hypothesis 2 was not supported. These findings are consistent with the results of another survey of teenagers. In this study, no moderating effect was observed from physical exercise on the relationship between stress and life satisfaction (4). The reason why the results are not significant may be that this study only discussed the buffering effect of physical exercise on stress symptoms. There is evidence supporting the mental stress buffering effect of adolescent physical exercise when other variables are considered, such as negative emotions, including depression and dissatisfaction, and positive emotions, such as feeling happy (20). Therefore, physical exercise may affect stress symptoms less than mood. This result suggests that physical exercise among adolescents may not fully buffer the stress symptoms resulting from increased mental stress events during an epidemic. Additional amplified or weakened regulatory factors may also affect the buffering effect of physical exercise on the relationship between mental stress events and stress symptoms. The mental stress buffer effect may not be significant if these regulatory factors are not considered in the relationship model. Thus, we hypothesized that health motivation might regulate the buffering effect of physical exercise on mental stress during an epidemic.

### 5.2. Regulation of health motivation on the mental stress buffering effect of physical exercise of adolescents during the epidemic

The mental stress buffering effect was only increased when adolescents engaged in high levels of moderate exercise with high health motivation, supporting Hypothesis 3. Therefore, health motivation is an important factor requiring special attention during an epidemic. When trying to buffer mental stress through physical exercise, we should advocate cultivating and stimulating health motivation.

During the epidemic, adolescents’ health motivation for exercise was significantly positively correlated with physical exercise. Li et al. (21)‘s results support this view, indicating that the promotion effect of health motivation on college students’ physical exercise is greater than other types of motivation. Li et al. attributed health motivation to external motivation, suggesting that external motivation can better promote health behavior. Litt et al. (22) investigated the exercise motivation of 9,011 adolescents, and the results showed that health-related external motivation has a stronger impact on physical exercise among adolescents. Kilpatrick et al. (23) found that the main motivation of female college students to exercise is to improve their appearance, while the main motivation of male college students is to improve their social interaction levels. Although the types of effective motivation in each study differ, it can be inferred that the motivation more closely related to individual needs may be more decisive. Therefore, because epidemics like COVID-19 pose a great threat to health (24), health motivation may be a key variable in promoting physical exercise among adolescents in such scenarios.

The correlations between adolescent health motivation and physical exercise and mental stress events and stress symptoms were significant during the epidemic and regulated physical exercise-related mental stress buffering. When the level of health motivation is high, the mental stress buffering effect of physical exercise is stronger. Physical exercise during an epidemic helps cultivate physical fitness, immunity, and an improved emotional state, buffering mental stress (3). Therefore, when an individual is motivated to obtain health benefits from physical exercise, this motivation could help to buffer the mental stress of physical exercise (25). Ntoumanis et al. (26) found that physical exercise can positively impact health when it is considered to increase personal utility. The results of this study emphasized the importance of health motivation and the limitations associated with epidemics. Health concerns due to viral infections and illness have become common and important stressors as a result of the COVID-19 pandemic. Health motivation and the desire to increase health *via* physical exercise is a potential coping mechanism adopted by the public that can increase the possibility of benefiting from physical exercise through mental stress buffering.

However, some evidence is in contrast with the view of this study. It has been stated based on the theory of self-determination—which states that internal motivation encourages participation in behaviors due to self-interest—that individuals have a universal need for autonomy and self-determination (27). Internal motivation is more likely to promote individual behavior participation (28). Internal motivation is based on individual needs and helps overcome difficulties in regular or long-term behaviors; thus, internal motivation is considered the key factor affecting participation in physical exercise (29). In the study of physical exercise, the satisfaction of internal motivation is often accompanied by positive emotional and subjective well-being improvement, which is more likely to form the effect of a mental stress buffer (30). This effect may differ depending on the situation. Internal motivation is considered the only predictor of physical exercise engagement among adolescents in normal situations (31). During the epidemic, due to the lack of specific drugs for COVID-19, the negative news of infectious diseases, severe diseases, and deaths constantly affected individuals, creating a situation different from the norm in which people paid more attention to their health (31). Thus, in an epidemic, individuals may pay more attention to health than interest. Because physical exercise has been widely recognized as a behavior beneficial to health, in the context of special attention to health, individual physical exercise is beneficial to obtain positive emotions such as comfort and pleasure, which is more likely to be the key factor affecting the mental stress buffer effect. However, with the improvement of the epidemic situation in the future, people’s health motivation may decrease. Therefore, attention should be paid to the role of internal motivation in the mental stress buffering effect of physical exercise—especially the transformation from external motivation to internal motivation—to better promote physical exercise and its physical and psychological benefits (31).

Separating physical exercise by high and moderate volumes in the context of health motivation revealed a significant regulatory effect on the mental stress buffering effect of a moderate volume of exercise; however, no effect was observed with a large volume of exercise. The existing meta-analysis evidence for using physical exercise for depression supports this view. Moderate-intensity physical exercise can alleviate depression, while continuous high-intensity physical exercise may induce stress and aggravate individual depression symptoms (32). Clark et al. (33) summarized the impact of physical exercise on mental stress and showed that long-term vigorous physical exercise (VO2 max ≥60–70%) does not relieve mental stress but induces typical stress symptoms. The concentration of cortisol, epinephrine, and norepinephrine is increased due to heavy physical exercise. To redistribute oxygen to the working muscles, the blood flow in the viscera and other organs is reduced. The subsequent decrease in the intestinal epithelial blood supply and reperfusion can lead to hypoxia, acidosis, ATP depletion, free radical formation, and oxidation/nitrification stress. These factors jointly diminish the intestinal barrier, resulting in increased intestinal permeability (34). Subsequently, LPS/endotoxin translocates to the circulatory system, resulting in an insufficient supply of intestinal blood, nutrients, water, and oxygen, and inducing typical stress symptoms, such as inflammatory reactions and gastrointestinal discomfort (35). In contrast, the cross-stressor adaptation hypothesis states that high-intensity physical exercise associated with strong competition and fierce resistance effectively stimulates the body to adapt to mental stress (36). However, during the COVID-19 epidemic, adolescents had existing high mental stress levels; thus, high-intensity exercise may be insufficient to cultivate the body’s coping ability. Moderate-intensity physical exercise is, therefore, more suitable as an intervention method for teenagers with existing stress. Von Haaren et al. (37) found that long-term, regular, moderate, and moderate-intensity yoga and other physical activities are more suitable for individuals with high stress levels. Yang Jian et al. (38) also found that moderate physical exercise is a necessary and effective means to relieve mental stress in teenagers. Therefore, moderate exercise as a mental stress buffer is recommended during an epidemic.

## 6. Conclusion

Through a nationwide sampling survey during the epidemic, this study found a close relationship between adolescent mental stress events and stress symptoms. It showed that the mental stress buffering effect is found only when adolescents engage in a high level of moderate physical exercise due to high health motivation. Therefore, in the post-epidemic period, physical exercise is recommended to alleviate stress in adolescents; however, it is also necessary to control the intensity and duration of physical exercise and focus on moderate-intensity physical exercise. Furthermore, the impact of physical exercise on health is crucial and exercise-related health motivation should be stimulated in teenagers.

## Data availability statement

The raw data supporting the conclusions of this article will be made available by the authors, without undue reservation.

## Ethics statement

The studies involving human participants were reviewed and approved by Ethics Committee of Nantong University (2022) No. 70. Written informed consent to participate in this study was provided by the participants’ legal guardian/next of kin.

## Author contributions

HL: manuscript writing, study design, and manuscript revising. JC: data analysis and manuscript revising. PL: manuscript revising and data analysis. All authors contributed to the article and approved the submitted version.

## Funding

This work was supported by general program of Education of the National Social Science Fund of China: “Research on sports regulation mechanism and intervention scheme of middle school students’ psychological pressure.” (BLA210215).

## Conflict of interest

The authors declare that the research was conducted in the absence of any commercial or financial relationships that could be construed as a potential conflict of interest.

## Publisher’s note

All claims expressed in this article are solely those of the authors and do not necessarily represent those of their affiliated organizations, or those of the publisher, the editors and the reviewers. Any product that may be evaluated in this article, or claim that may be made by its manufacturer, is not guaranteed or endorsed by the publisher.
